# Medical Statistics

**Published:** 1837-04

**Authors:** 


### MEDICAL STATISTICS.

1. *On a Method of determining the Danger and the mean future Duration of Diseases at different Periods of their Progress.* 2. *On the Law of Recovery and Dying in Small-Pox.* By WILLIAM FARR, Esq.

THESE two papers are on subjects of no less difficulty than importance, and seem to open a path of extreme interest in medical science, but which, we fear, can be trodden at present by few but the learned author. As any extracts from these memoirs cannot give a just idea of their value, and as we doubt our own capacity to do them justice in the form of an abstract, we must content ourselves by giving, from the first paper, a brief note of the early steps in the process, referring our readers for its complete development to the original.

It is an ascertained fact, that, when large numbers of patients are considered in the same circumstances, nearly an equal proportion will invariably die; that, if, out of 5000 patients labouring under fever, 500 die at one time and 4500 recover, nearly the same proportion of similar cases will perish at another time.

At the origin of the disease, the mean possibility of recovery would be, in the instance cited, as 4,500 to 500; for 4,500 would recover, and 500 would die: or, in other words, out of 5,000 chances, 500 would be in favour of death, 4,500 in favour of recovery. Where the mortality in a disease is ten per cent., it is nine to one in favour of recovery; the probability of recovery is expressed by the decimal .9, or by the vulgar fraction $\frac{9}{10}$.

This principle holds at every period of the disease: if it be known that, of the patients who survive the twentieth day, 3,268 recover, 300 die, the chance of recovery at the end of the twentieth day is as 3,268 to 300, &c.

By a very simple enumeration and arrangement, the number that survive, die, or recover after every day,—every fifth, tenth, or any other day,—can be determined. For example, it was ascertained that, out of 4,915 cases of small-pox, at the end of five complete days, 149 died in the next five days, or between the fifth and tenth day: consequently, only 4,766 cases remained at the end of ten complete days; and of these 4,766 cases, 1,452 terminated fatally. There were 4,766 chances at the end of the tenth day; 1,452 in favour of death, and the rest, 3,314, in favour of recovery. The first step in the enquiry, therefore, is to determine, from a proper registry, the number of cases terminating every five days, (a day, week, or any other period, may be employed,) distinguishing the fatal from the other cases. This is done in Table II. of the present article.

*British Annals of Medicine, Jan. 20th and Feb. 3d,* 1837.

